# Treatment resistant M-type phospholipase A2 receptor associated membranous nephropathy responds to obinutuzumab: a report of two cases

**DOI:** 10.1186/s12882-022-02761-3

**Published:** 2022-04-07

**Authors:** Rebecca Hudson, Cassandra Rawlings, Saw Yu Mon, Julia Jefferis, George T. John

**Affiliations:** 1 Kidney Health Service, Royal Brisbane and Women’s Hospital, Level 9 Ned Hanlon Building, Butterfield Street, Herston, Queensland 4029 Australia; 2 Department of Renal Medicine, Townsville University Hospital, Douglas, QLD Australia; 3 Faculty of Medicine, University of Queensland, Brisbane, Australia

**Keywords:** Obinutuzumab, Membranous nephropathy, Resistant

## Abstract

**Background:**

Membranous Nephropathy (MN) is a common cause of nephrotic syndrome (NS) in adults. Recognition of MN as an antibody mediated autoimmune disease has enabled the introduction of anti-B-cell therapy. Rituximab, a type I anti-CD20 antibody has been used in the management of MN, but has a 35-45% failure rate. Obinutuzumab, a fully humanised type II anti-CD20 monoclonal antibody produces greater CD20 depletion and is superior to rituximab in the treatment of certain B-cell malignancies. In the two reports published to date involving nine patients with M-type phospholipase A2 receptor (PLA2R) associated MN (six of whom were rituximab resistant), treatment with obinutuzumab lead to immunological remission (IR) in 75% of patients, with improvement of proteinuria, normalisation of serum albumin and stable renal function in all patients.

**Case presentation:**

We report on two cases of PLA2R-associated MN, two males aged 33 and 36-years, who presented with NS and bilateral sub massive pulmonary emboli requiring anticoagulation. Both were diagnosed serologically as PLA2R-associated MN where a renal biopsy was initially deferred due to bleeding risk on anticoagulation, but later confirmed. Both patients were refractory to multiple lines of therapy including rituximab, but achieved IR, normalistation of serum albumin, improved proteinuria and stable renal function with obinutuzumab.

**Conclusions:**

Our cases add to the current limited literature on the successful use of obinutuzumab in PLA2R associated MN refractory to standard therapy including rituximab.

**Supplementary Information:**

The online version contains supplementary material available at 10.1186/s12882-022-02761-3.

## Background

Membranous nephropathy (MN) is a common cause of non-diabetic nephrotic syndrome (NS) in white adults [1, 2]. Left untreated 30-50% of patients with persistent NS may progress to end stage kidney disease over ten years [1, 3, 4]. Up to 70% of patients with MN have autoantibodies to M-type phospholipase A2 receptor (PLA2R) in the serum [5]. Recognition that B-cells play an integral part in the pathogenesis of MN has enabled the development and use of targeted anti-B-cell therapy. Rituximab, a type I anti-CD20 antibody, is an effective treatment for patients with idiopathic MN with 55-65% of patients achieving complete or partial remission [2, 6], however this leaves a significant failure rate. There is growing evidence that immunological remission (IR) precedes improvement in proteinuria [7, 8]. Obinutuzumab® (Gazyza [Genentech, South San Francisco SA]) is a humanised type II anti-CD20 monoclonal antibody directed at a different epitope on CD20 than engaged by rituximab yielding profound B-cell apoptotic response and CD20 depletion [9].

We describe two patients with PLA2R associated MN treated successfully with obinutuzumab where standard therapy including rituximab failed to induce immunological and clinical remission. The definitions of immunological, complete and partial remission are in the supplementary document [10].

## Case presentation

### Case one

**Fig. 1:**
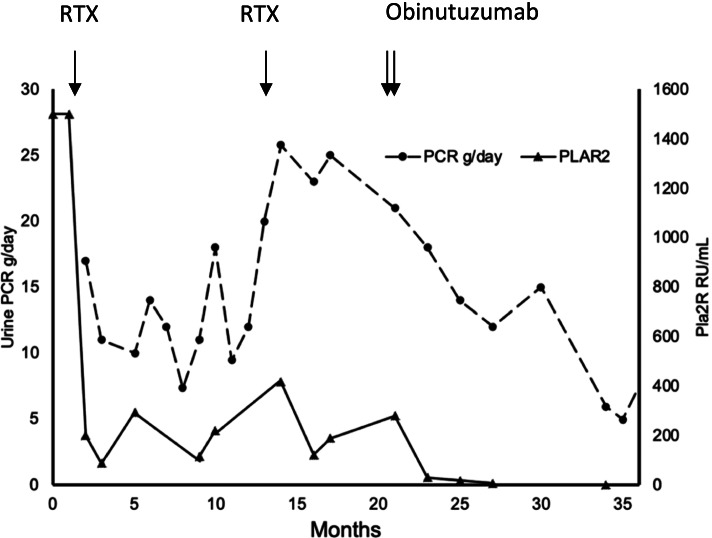
Clinical course, proteinuria, PLA2R antibody level and response in relation to anti-CD20 treatments in case 1. RTX, rituximab; PCR, protein creatinine ratio; PLA2R, phospholipase A2 receptor

A 36-year-old white male presented with NS and bilateral sub massive pulmonary emboli requiring anticoagulation. Past medical history included hypertension on ramipril 2.5mg daily, obesity (131.7kg) and sacroiliitis with iritis, with no other medications or drug use. He was diagnosed serologically as PLA2R-associated MN. At presentation, laboratory testing showed a PLA2R antibody titer of >1500 RU/mL, urine protein creatinine ratio (PCR) of 17g/day, urine leucocytes of 30 x 10^6^/L and red cells 40x10^6^/L without epithelial cells, serum albumin of <15g/L and serum creatinine (Scr) of 56umol/L (Fig. 1). Secondary screening for viruses (hepatitis B [HBV] and C [HCV] and human immunodeficiency [HIV]), autoimmune disease (specifically antinuclear antibody [ANA]) and malignancy (including a full body computerised tomography [CT] scan) were negative. Imaging revealed a renal vein thrombosis in the absence of renal cancer, while a thrombophilia screen including lupus anticoagulant, anti-cardiolipin antibodies and anti-beta 2 glycoprotein antibodies was negative. A renal biopsy was deferred because of high bleeding risk and renal vein thrombosis. The ramipril 2.5mg was unable to be titrated and required cessation due to recurrent acute kidney injury. He was managed with two doses of rituximab (1g each) intravenously (IV) fortnightly. Three months after rituximab the PLA2R antibody titre reduced to 295 RU/ml, PCR decreased to 10g/day, serum albumin remained <15g/L and Scr increased to 90umol/L with early repopulation of CD19 count to 0.08 x10^9/L and recanalisation of the thrombosed renal vein. On account of the partial response, he was treated with cyclosporine 300mg twice daily. Subsequently, to contain severe nausea, cyclosporine doses were reduced with a target trough level of 75-125ug/L. The laboratory parameters worsened; PLA2R antibody titre remained elevated at 220 RU/mL, PCR increased to 18g/day, serum albumin remained <15g/L and Scr increased to 144umol/L. He received IV cyclophosphamide 1200mg with prednisolone 75mg daily. Following the fifth cycle of cyclophosphamide PLA2R antibody titer increased to 417 RU/mL, PCR increased to 25.79g/day, with a serum albumin of 22g/L and Scr of 149umol/L. Cyclophosphamide was stopped and prednisolone weaned to 7.5mg over six months. He then received two more doses of rituximab (1g each) fortnightly and six months later the PLA2R antibody titer was 279RU/mL, the PCR was 21g/day, the serum albumin was <15g/L and Scr increased to 261umo/L, with a CD19 cell count of 0.01 x10^9/L. A renal biopsy showed MN with PLA2R staining with a low total renal chronicity score of 2/10 and 8% globally sclerosed glomeruli. He received IV obinutuzumab100mg, 900mg and 1g on days one, two and sixteen respectively with pre-medications of 20mg IV dexamethasone and 10mg oral loratadine. One year after treatment, PLA2R antibody titer was undetectable at <2.1RU/mL, PCR reduced to 5.94g/day, serum albumin increased to 33g/L and Scr improved to 164umol/L with a suppressed CD19 cell count of 0.02 x10^9/L. At time of last follow up, at fifteen months he remained in IR with an undetectable PLA2R antibody titer, proteinuria of 7.56g/day and normalisation of serum albumin to 42g/L, and improved renal function with a Scr of 113umol/L and an eGFR of 72ml/min.

### Case two

**Fig. 2:**
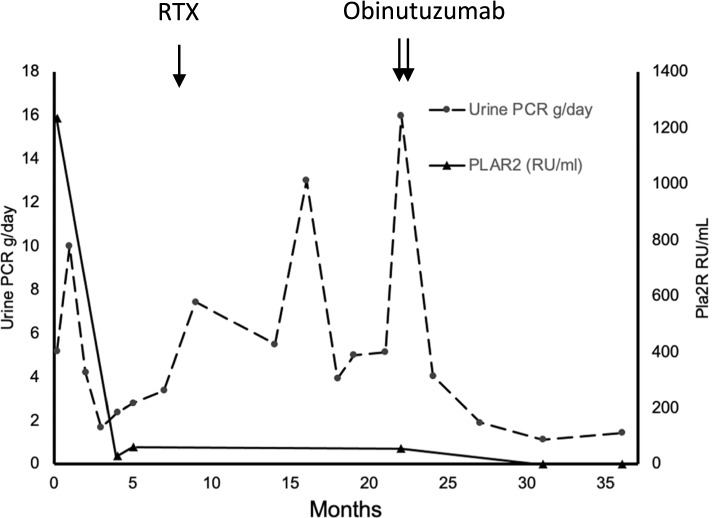
Clinical course, proteinuria, PLA2R antibody level and response in relation to anti-CD20 treatments in case 2. RTX, rituximab; PCR, protein creatinine ratio; PLA2R, phospholipase A2 receptor

A 33-year-old white male presented with NS and associated bilateral sub massive pulmonary emboli, diagnosed serologically as PLA2R-associated MN. Past medical history included recurrent provoked venous thromboembolic disease, without any identified cause and on no current therapy prior to presentation. Secondary screening for viruses (HBV, HCV and HIV) and autoimmune disease (specifically ANA) were negative. Malignancy screen including a full body CT scan and a positron emission tomography scan which were both negative, however; immunofixation identified a kappa immunoglobulin A band in trace amounts which was further investigated with a bone marrow aspirate with less than 5% plasma cells, and attributed to a monoclonal gammopathy of unclear significance, for surveillance only. Imaging revealed a renal vein thrombosis in the absence of renal cancer, while a thrombophilia screen including lupus anticoagulant, anti-cardiolipin antibodies and anti-beta 2 glycoprotein antibodies was negative. At presentation laboratory testing showed a PLA2R antibody titer of 1235 RU/mL, urine PCR of 5.17 g/day, urine sediment with 20 x 10^6^/L white cells, <10 x 10^6^/L erythrocytes and epithelial cells, serum albumin of 22g/L and Scr of 88umol/L (Fig. 2). Biopsy of the horseshoe kidney was deferred while on anticoagulation. He was treated with perindopril titrated to maximum tolerated dose (8mg), in addition to receiving cyclosporine 200mg twice daily with trough level target of 150-250ug/L and prednisolone 15mg daily, with a decline in PLA2R titre to 29 RU/mL at three months, which increased to 62 RU/ml at four months. He received a total of seven months of cyclosporine, following treatment PCR was 3.37g/day, serum albumin, 27g/L and Scr 87umol/L, the PLA2R antibody was not retested. He was then treated with two doses of IV rituximab (1g each) fortnightly. Six months following rituximab, PLA2R was still detected but not quantified, PCR increased to 5.5 g/day, serum albumin declined to 18g/L with a Scr of 73umol/L. He was treated with oral cyclophosphamide 100mg daily in addition to prednisolone 5mg daily, with a cumulative dose of 5g of cyclophosphamide. MN remained refractory with a PLA2R antibody titre of 55RU/mL, PCR of 5.08g/day, serum albumin of 24g/L and Scr of 85umol/L. A renal biopsy confirmed MN, staining positive for PLA2R with a chronicity score of 0/10. The patient received IV obinutuzumab 100mg, 900mg and 1g on days one, two and twenty-one respectively with pre-medications of 20mg IV dexamethasone and 10mg oral loratadine. Nine months after receiving obinutuzumab, PLA2R antibody titre was undetectable at <2.1RU/mL, PCR reduced to 1.12g/day, serum albumin normalised at 36g/L, with a preserved Scr of 80umol/L and suppressed CD19 cell count of 0.03 x10^9/L. At twelve months PLA2R antibody titer remained undetectable, PCR 1.43g/day, serum albumin 43g/L and Scr 85umol/L with an eGFR of >90ml/min.

Neither patient experienced infusion-related side effects or infectious complications following treatment with obinutuzumab (Gazyza®, Roche) costing AUD $5,293 per 1000mg vial.

## Discussion and conclusions

We present two cases of PLA2R-associated MN refractory to prednisolone, cyclosporine, cyclophosphamide and rituximab but subsequently responded to obinutuzumab. They showed IR and normalisation of serum albumin and stable renal function. Both had lower levels of proteinuria persisting presumably from the partially sclerosed glomeruli. Residual proteinuria in MN has been shown in animal models to be secondary to remodelling of the glomerular basement membrane resulting in altered architecture of the podocytes [11].

Both patients at presentation had significant proteinuria (5.5-25.79g/day) and received rituximab following which both saw a reduction in titres of PLA2R antibodies but without IR. There is evidence that IR heralds improvement in proteinuria [7, 8]. The lack of response to rituximab may be from incomplete B-cell depletion. Obinutuzumab has an increased antibody dependent cell cytotoxicity in comparison to a more complement mediated activity in rituximab, in addition to a more profound CD20 depletion, and lower risk of immunogenicity [12, 13]. Obinutuzumab is likely to be more effective than rituximab in certain B-cell malignancies such as chronic lymphocytic leukaemia and follicular lymphoma [13, 14], in addition, it has also been shown to reduce CD19 counts in sensitised kidney transplant candidates in a small trial [15].

Both patients responded to obinutuzumab. These findings confirm the limited evidence regarding the benefit of obinutuzumab in idiopathic MN. Included is a case series of three patients with rituximab refractory PLA2R-associated MN where treatment with obinutuzumab lead to IR in two patients; improvement of proteinuria, normalisation of serum albumin and stable renal function in all three patients (Table 1; cases 3-5) [16]. Two patients received 2g of obinutuzumab and reached IR, one patient received 1g and did not reach IR. Two of the three patients had a sustained partial remission at eighteen and twenty-four months, with improved proteinuria in the third patient at nine months. The second report is a single-centre cohort study of ten patients with refractory MN (six of whom were PLA2R positive, and three of whom had received rituximab) where treatment with 2g of obinutuzumab resulted in 40% complete and 50% partial remission, with all patients who were serum PLA2R positive achieving IR (Table 1; cases 6-11) [10]. Those who achieved remission by twelve months and had longer follow up, maintained it at twenty-four months [10]. Renal function remained stable in all patients throughout treatment. The sustained response to obinutuzumab is reflected in our cases at last follow up, with the first patient maintaining IR at fifteen months and the second, at twelve months (Table 1; cases 1-2).

**Table 1:** Summary of the published cases to date of patients who have received obinutuzumab

Case	Age and Gender	Months post obinutuzumab	Initial PCR (g/day)	Final PCR (g/day)	Initial creatinine umol/L	Final creatinine umol/L	Initial serum albumin g/L	Final serum albumin g/L	Initial PLA2R titer (RU/mL)	Final PLA2R titer (RU/mL)	IR	Treatments Trialled prior to obinutuzumab
1	36M	15	17	7.56	56	113	<15	42	>1500	<2.1	Yes	Rituximab 4g total, cyclosporine, prednisolone, cyclophosphamide
2	33M	12	5.17	1.43	88	85	22	43	1235	<2.1	Yes	Rituximab 2g total, cyclosporine, prednisolone, cyclophosphamide
3	54F	18	8.6	1.1	195	115	33	43	312	5	No	Rituximab 4g total
4	61M	9	21	6.8	97	124	20	38	100	<2	Yes	Rituximab 2g total, cyclosporine, prednisolone, cyclophosphamide
5	54M	24	19.7	1.5	177	150	22	41	170	<2	Yes	Rituximab 4g total
6	66M	6	11.3	*5.9	186	*140	17	*20	633	5.1	Yes	Rituximab
7	41M	24 (PLA2R last tested at 12 months)	10.73	*2.2	80	*71	27	*37	39	1.8	Yes	Prednisolone, tacrolimus
8	68M	36	7.8	*0.5	115	*106	21	*38	261	NA	NA	Rituximab
9	76F	12	5.8	*0.5	124	*106	28	*35	79	3.2	Yes	Prednisolone, tacrolimus
10	67M	6 (died at 6 months)	8.49	*3.2	133	*97	28	*32	57	7.6	Yes	Rituximab, prednisolone, tacrolimus, mycophenolate
11	50M	6	4.6	*1.2	133	*133	36	*38	NA	NA	NA	Prednisolone, tacrolimus, mycophenolate

Cases 1 and 2 are from the current case report.

Cases 3-5 published by Klomjit et al.

Cases 6-11 published by Sethi et al.

Case 8 reached IR without improved proteinuria with rituximab, as such had a normal PLA2R antibody titer at time of obinutuzumab therapy.

Case 11 was serum PLA2R negative but stained positive in their renal biopsy specimen.

*data obtained from the graph of the paper by Sethi et al without the raw data available.

*M* male, *F* female, *PCR* protein creatinine ratio, *PLA2R* phospholipase A2 receptor, *IR* immunological remission, *NA* not available.

In summary, this case report of benefit with obinutuzumab adds to the previous two reports of its efficacy. Though the long-term efficacy and impact remain unclear, obinutuzumab is being increasingly considered for PLA2R associated MN resistant to standard therapy. A phase III randomised controlled trial evaluating the efficacy and safety of obinutuzumab compared to tacrolimus in patients with primary MN is currently under way (NCT04629248).

## Supplementary Information

**Additional file 1.**

## Data Availability

The data used during the current study are available from the corresponding author on reasonable request.

## Abbreviations

MN: Membranous nephropathy
NS: Nephrotic syndrome
PLA2R: Autoantibodies to M-type phospholipase A2 receptor
IR: Immunological remission
PCR: Protein creatinine ratio
Scr: Serum creatinine
IV: Intravenously
HBV: Hepatitis B
HCV: Hepatitis C
HIV: Human immunodeficiency virus
ANA: Antinuclear antibody
CT: Computerised tomography
eGFR: Estimated glomerular filtration rate
AUD: Australian dollars
RTX: Rituximab
M: Male
F: Female
NA: Not available
