# Supplementary material for: Treatment resistant M-type phospholipase A2 receptor associated membranous nephropathy responds to obinutuzumab: a report of two cases
Source: BMC Nephrol. 2022 Apr 7;23:134. doi: 10.1186/s12882-022-02761-3 (PMC8991934; doi:10.1186/s12882-022-02761-3)
Supplement: Supplementary file 1 — Additional file 1. [file 12882_2022_2761_MOESM1_ESM.docx]

**Supplementary material**

**Definitions**

**Immunological remission (IR);** defined as an undetectable PLA2R antibody titer by enzyme-linked immunosorbent assay, where values of <14 RU/mL ^[1]^ are considered negative by the manufacturer, but some studies have shown that levels 2-14 RU/mL can be detected with other assays. In the case report by Klomjit et IR was a defined as a PLA2R antibody titer <2 RU/mL ^[2].^ Whereas, in the study by Sethi et al, the definition of IR was a PLA2R antibody titer of <14 RU/mL ^[3].^

We used a definition of PLA2R antibody titer of <2.1 RU/mL as IR as this is the cut off target titer on our labs assay.

**Complete Remission;** defined in the MENTOR trial as urine protein creatinine ratio of <0.3g/24h and serum albumin >3.5g/dl ^[4]^. In the study by Sethi et al, complete remission was defined as a urine protein creatinine ratio of ≤ 0.3 g/g and a stable serum creatinine(within 25% of baseline) ^[3].^

We defined complete remission as urine protein creatinine ratio of ≤ 0.3 g/d, serum albumin >35g/L and a stable serum creatinine (within 25% of baseline).

**Partial remission;** defined in the MENTOR trial as reduction in proteinuria of >50% from baseline up to 3.5g/24h but >0.3g/24h ^[4].^

In the study by Sethi et al, partial remission was defined as urine protein creatinine ratio of between 0.3 g/g and 3.5g/g and a stable serum creatinine ^[3].^

We defined partial remission as urine protein creatinine ratio of between 0.3 g/d and 3.5g/d and a stable serum creatinine.

1. Ruggenenti, P., et al., *Anti-Phospholipase A2 Receptor Antibody Titer Predicts Post-Rituximab Outcome of Membranous Nephropathy.* J Am Soc Nephrol, 2015. **26**(10): p. 2545-58.

2. Klomjit, N., F.C. Fervenza, and L. Zand, *Successful treatment of patients with refractory PLA2R-associated membranous nephropathy with obinutuzumab: a report of 3 cases.* American Journal of Kidney Diseases, 2020. **76**(6): p. 883-888.

3. Sethi, S., et al., *Obinutuzumab is Effective for the Treatment of Refractory Membranous Nephropathy.* Kidney international reports, 2020. **5**(9): p. 1515-1518.

4. Fervenza, F.C., et al., *A Multicenter Randomized Controlled Trial of Rituximab versus Cyclosporine in the Treatment of Idiopathic Membranous Nephropathy (MENTOR).* Nephron, 2015. **130**(3): p. 159-68.
